# Guideline clinical nutrition in patients with stroke

**DOI:** 10.1186/2040-7378-5-14

**Published:** 2013-12-01

**Authors:** Rainer Wirth, Christine Smoliner, Martin Jäger, Tobias Warnecke, Andreas H Leischker, Rainer Dziewas

**Affiliations:** 1 Department of Internal Medicine and Geriatrics, St. Marien-Hospital Borken, Am Boltenhof 7, D-46325 Borken, Germany; 2 Institute for Biomedicine of Aging (IBA), Chair of Geriatric Medicine, Friedrich-Alexander-University Erlangen-Nürnberg, Erlangen, Germany; 3 Department of Geriatrics, St. Vinzenz-Hospital, Dr.-Otto-Seidel-Strasse 31-33, D-46535 Dinslaken, Germany; 4 Department of Neurology, University Hospital Münster, D-48129 Münster, Germany; 5 Department of Internal Medicine, Oncology and Geriatrics, Alexianer-Hospital, Dießemer Bruch 81, D-47805 Krefeld, Germany

**Keywords:** Dysphagia, Guideline, Stroke, Tube feeding

## Abstract

Stroke is regularly accompanied by dysphagia and other factors associated with decreased nutritional intake. Dysphagia with aspiration pneumonia and insufficient nutritional intake lead to worse outcome after stroke.

This guideline is the first chapter of the guideline “Clinical Nutrition in Neurology” of the German Society for Clinical Nutrition (DGEM) which itself is one part of a comprehensive guideline about all areas of Clinical Nutrition. The thirty-one recommendations of the guideline are based on a systematic literature search and review, last updated December 31, 2011. All recommendations were discussed and consented at several consensus conferences with the entire DGEM guideline group. The recommendations underline the importance of an early screening and assessment of dysphagia and give advice for an evidence based and comprehensive nutritional management to avoid aspiration, malnutrition and dehydration.

## Introduction

In the acute stage of stroke 30 to 50% of patients suffer from dysphagia, while the incidence drops to around 10% six months later. Affected patients are not only prone to dehydration and malnutrition but are also at an increased risk of acquiring aspiration pneumonia. Several studies have shown the risk for this complication being up to 12-fold increased in dysphagic stroke patients and occurring in up to 30% of patients in certain patient groups [1-5]. Mainly because of this potentially life-threatening complication, morbidity and mortality are significantly increased in dysphagic stroke patients as opposed to non-dysphagic stroke victims [6,7]. Due to its prognostic importance, an early detection of stroke related dysphagia and a suitable nutritional management is therefore of utmost clinical importance.

## Methodology

Authors are representatives of three German medical societies: the German Society for Clinical Nutrition (DGEM), the German Society for Neurology (DGN) and the German Geriatric Society (DGG). All authors worked on an honorary basis. Travelling expenses for the meetings were covered by the DGEM. Six working group meetings were performed between October 2010 and June 2012. The literature search was last updated December 31, 2011. The following databases were screened for relevant literature: Medline/PubMed, National Institute for Health and Clinical Excellence (http://www.nice.org.uk), Scottish Intercollegiate Guideline Network (http://www.sign.ac.uk) and Google (http://www.google.com).

The following types of publications were screened for relevance: randomized controlled trials, cohort studies, case–control studies, cross-sectional surveys, systematic reviews, meta-analyses and guidelines.

For this part of the guideline the following terms were included to the literature search: stroke AND nutrition NOT prevention; intracerebral bleeding AND nutrition; stroke AND perc*; stroke AND endosc*; stroke AND gastr*; stroke AND tube feeding; stroke AND enteral feeding; stroke AND nutritional supplements*; stroke AND oral supplement*; stroke AND aspiration; stroke AND dysphagia; stroke AND malnutrition; stroke AND undernutrition; stroke AND swallowing; stroke AND infections; stroke AND gastric motility; nutrition AND rehabilitation; nutrition AND pressure sores; nutrition AND quality of life; nutrition AND infections; nutrition AND aspiration.

Evidence levels of every publication were adapted from AHCPR [8] and were graded as follows:

Ia: Evidence obtained from meta-analysis of randomized controlled trials (RCTs)

Ib: Evidence obtained from at least one randomized controlled trial (RCT)

IIa: Evidence obtained from at least one well designed controlled study without randomisation

IIb: Evidence obtained from at least one well designed “quasi-experimental” study

III: Evidence obtained from well-designed non-experimental descriptive studies such as comparative studies, correlation studies and case studies

IV: Evidence obtained from expert committee reports and/or opinions or clinical experience of respected experts on the field

The evidence grade of recommendations was suggested by the working group based on the evidence available and the clinical relevance. The proposal of the working group was presented to an anonymous internet based voting procedure with all guideline working group members of DGEM. If the agreement of the internet voting was less than 95%, the suggested recommendations were discussed, adapted and again anonymously voted during two consensus conferences. Recommendations with scarce evidence, which however are important for clinical routine could be upgraded one level on decision of the consensus conference. The grading of recommendations was adapted from AHCPR [8].

### Grade A (good evidence)

Requires at least one randomized controlled trial as part of a body of literature of overall quality and consistency addressing the specific recommendation.

### Grade B (fair evidence)

Requires the availability of well conducted clinical studies but no randomized clinical trials on the topic of the clinical recommendation.

### Grade C (poor evidence)

Requires evidence obtained from expert committee reports or opinions and/or clinical experience of respected experts. Indicates the absence of directly applicable clinical studies of good quality.

### CCP = clinical consensus point

Decision of the consensus conference, due to strong clinical evidence without evidence from clinical trials. The methodological approach is extensively described by the association of German scientific medical societies (AWMF) [9].

This guideline is a translation of the first chapter of the German DGEM-guideline “Clinical Nutrition in Neurology”, which is one chapter of the comprehensive DGEM-guideline “Clinical Nutrition”. In addition, it has to be mentioned that the short part about screening and assessment of the nutritional state was added from the previous version of the guideline (recommendation 10 and 11) [10], because the actual DGEM-guideline includes an entire chapter on nutritional screening and assessment, which is to extensive to be cited here and not specifically designed for stroke patients.

## Screening and assessment for dysphagia in stroke patients

### Which methods should be used for dysphagia screening? How should the risk of aspiration be evaluated?

#### Recommendation 1

A formalised screening for dysphagia should be performed in all stroke patients (B).

A formalised screening for dysphagia should be carried out in all acute stroke patients as part of the initial examination or upon arrival of the patient on the hospital ward/stroke unit, i.e. normally within a few hours after hospital admission. The following three methods have been evaluated in acute stroke patients and may be considered.

**Water-Swallowing-Test (WST).** Several different protocols have been suggested and published with the main difference being the amount of water chosen for the swallowing screening [11-17]. Based on the SIGN-guidelines, a 50 ml-WST may be recommended for the use in daily routine.

In case that clinical signs of aspiration occur during the testing, the WST is considered positive. Due to the overt risk of aspiration, the patient is kept nil by mouth and more sophisticated diagnostic procedures are initiated (see below). In case the patient passes the WST, oral feeding may be started, although concrete dietary recommendations are not deducible from this test. Therefore patients should continuously be observed during feeding for the occurrence of coughing and chest infection.

**Multiple-Consistency-Test.** Originally published as “Gugging Swallowing Screen (GUSS)”, this multiple-consistency-test has the important advantage over the WST that it results in detailed recommendations for dietary management [18]. The GUSS is designed as a stepwise procedure enabling a graded rating of dysphagia with separate evaluations for nonfluid and fluid textures. As a result of this test dysphagia is graded in one of four categories (severe, moderate, mild or no dysphagia). For each severity code a special diet and further strategies are recommended.

**Swallowing-Provocation-Test (SPT).** The swallowing-provocation-test (SPT) examines exclusively the involuntary swallowing reflex by bolus injection of 0.4 ml of distilled water through a small nasal catheter into the oropharynx. The SPT is considered normal if the time from water injection to reflexive swallowing is equal or below three seconds. If the swallowing reflex is delayed for more than three seconds, the test is abnormal and the patient is deemed to be at risk of aspiration.

During the last years, the importance of dysphagia screening in patients with acute stroke has been supported by different, methodologically heterogeneous studies. Several prospective observational studies showed associations between a pathological dysphagia screening and an increased incidence of pneumonia [19,20] as well as a reduction of infectious complications after implementation of a systematic screening [20,21]. Hinchey and co-workers found in a large prospective, multicenter, observational study (N = 2532) that acute care institutions with a formal dysphagia screening show lower rates of pneumonia and mortality than institutions without such a formal arrangement [22].

In spite of this evidence, the impact of bed-side dysphagia screening, in particular the accuracy of the WST (Water Swallow Test) has been repeatedly questioned during recent years. Two meta-analyses of Ramsey et al. and Bours et al. suggested that when compared to VFSS (videofluorosopic swallowing study) or FEES (fiberoptic endoscopic evaluation of swallowing) the sensitivity of the WST for detecting aspiration is markedly below 80% in nearly all reviewed studies [23,24]. This observation also applies to specificity and negative and positive predictive values [23,24].

The multiple-consistency test according to the GUSS protocol has been evaluated in one prospective study [18] and performed with a sensitivity of 100% and a specificity of 50% when compared to FEES. Therefore this test seems to be more accurate in detecting dysphagic stroke patients than all versions of the simple WST. The main disadvantage of the GUSS protocol consists in its low specificity due to which dietary recommendations may be more restrained and nasogastric tubes may be inserted more often than actually necessary.

Subsequent to two smaller and retrospective studies [25,26] the SPT was prospectively evaluated in a collective of acute stroke patients [27]. When compared to FEES, the SPT had a sensitivity of 74.1% and a specificity of 100% for detecting aspiration. Due to its moderate sensitivity the SPT should not be used as stand-alone screening tool. However, given its high specificity, the SPT may be used as complement to other screening tools.

Several authors have suggested that pulse oxymetry may provide a useful non-invasive method of bedside swallowing testing [28-32]. In recent times however, this assumption has been rebutted by several studies [33-36]. Therefore, pulse oxymetry, whether alone or in combination with a WST, is *not* recommended for bedside dysphagia screening in stroke patients.

Finally, it has been suggested that an impaired pharyngeal sensation may be a suitable predictor of aspiration risk in stroke patients [37]. However, there is only one older study, which featured some methodological limitations, in support of this approach [15]. Therefore, and in agreement with the conclusion of Bours and co-workers [24], assessment of pharyngeal sensation is not recommended as screening tool for stroke-related dysphagia.

### In which patients is assessment of dysphagia indicated?

#### Recommendation 2

All stroke patients failing the dysphagia screening should be evaluated with a more thorough assessment of swallowing function (B).

#### Recommendation 3

Stroke patients without pathological findings in the initial bedside dysphagia screening should be referred to a further swallowing assessment if other known clinical predictors of dysphagia are present, such as a severe neurological deficit, marked dysarthria or aphasia or a distinct facial palsy (B).

Due to the insufficient sensitivity of most published screening procedures or missing replication studies, stroke patients without pathological findings in the initial bedside testing should be referred to a further swallowing assessment if other known clinical predictors of dysphagia are present, such as a severe neurological deficit, marked dysarthria or aphasia or a distinct facial palsy [19,38,39].

### Which methods should the used for the assessment of dysphagia?

#### Recommendation 4

Clinical bedside assessment (CBA): The CBA may be performed by trained personnel, typically a speech language pathologist, according to a standardised protocol (C).

#### Recommendation 5

Instrumental assessment of dysphagia: The limitations of clinical testing, in particular insufficient detection of silent aspiration and poor information on the efficacy of an intervention imply that a reliable, timely and cost effective instrumental swallow evaluation might be useful in acute stroke patients. Both VFSS and FEES may be used to this end (C).

#### Recommendation 6

Assessment of dysphagia should be carried out as early as possible (CCP).

The CBA published by Logemann contains 28 items and has been tested for inter- and intra-rater reliability [40]. Alternatively, other standardized protocols may be taken into consideration.

VFSS has long been regarded as gold standard in the assessment of dysphagia. VFSS dynamically visualizes the oral, pharyngeal and esophageal phases of swallowing. By using non-ionic contrast agents the risk of pulmonary complications in patients at risk of aspiration is minimized [41]. VFSS provides a comprehensive assessment of swallowing, determining not only whether the patient is aspirating but also why. Furthermore, it allows for experimentation with different textures, postures and manoeuvres suggested to improve the safety and efficiency of the swallow [42]. Penetration and aspiration are ideally graded according to the rating scale of Rosenbek et al. [43].

FEES is an instrumental assessment of swallowing using a flexible nasolaryngoscope which is passed through the nares, over the velum into the pharynx. Recent studies suggest that in patients with acute stroke FEES is a safe, reliable and predictive tool of dysphagia assessment [44-46]. Main advantages of FEES over VFSS, in particular with regard to acute stroke patients, are i) that the assessment can be done at the bedside, ii) that severely handicapped and uncooperative patients may be readily examined, iii) that the lack of radiation exposure enables short-term re-evaluations, and iv) that the saliva of the patients is directly visualized [47,48]. The main disadvantage of FEES compared to VFSS is that not the whole swallowing act is covered and that intradeglutitively, for a short moment the endoscopic view is impaired by the so called “white-out” [49]. However, in spite of this weakness of FEES two recent studies suggest that FEES is more sensitive than VFSS in detecting residues, penetration and aspiration [50,51].

### How often should the assessment of dysphagia be repeated?

#### Recommendation 7

During the first days of illness the CBA can be repeated in dysphagic stroke patients on a daily basis. If dysphagia persists, CBA can be carried out thereafter at least twice per week and before discharge (C). If the CBA is indicative of an improvement or a worsening of swallowing function an additional instrumental assessment (either FEES or VFSS) can be considered (C).

#### Recommendation 8

If dysphagia persists after discharge, assessment can be done at least once per month for 6 months after stroke manifestation (C).

During the first two weeks after stroke a substantial improvement of dysphagia is seen in a high number of patients, in particular in those with supratentorial lesions [4,11,13,52]. On the other hand, stroke recurrences, which are seen in 5 to 10% of patients within the first weeks [53], may cause a worsening of swallowing function. Therefore, a regular dysphagia assessment is necessary in acute stroke patients. From the therapeutical perspective, early initiation of swallowing rehabilitation is also indicated. Thus, Carnaby et al. have shown in their prospective randomised study, that early behavioural swallowing intervention was associated with a marked reduction of infectious complications and a significant increase of the proportion of patients regaining swallowing function [54].

### Which kind of grading should be provided by the dysphagia assessment?

#### Recommendation 9

Dysphagia assessment cannot be restricted to categorically observing whether dysphagia is present or absent but can provide a graded evaluation of dysphagia severity. Dysphagia assessment can be directly linked to appropriate protective and rehabilitative measures and can systematically offer nutritional recommendations (C).

Grading dysphagia is the prerequisite for a differentiated dysphagia management covering both protective and rehabilitative measures. Apart from that, it offers a reliable initial finding allowing to determine changes of the patient’s swallowing ability during the further clinical course [18,44,55]. Depending on the method of dysphagia assessment chosen there are different scales to be taken into consideration, for example the GUSS, or the FEDSS (Fiberoptic Endoscopic Dysphagia Severity Scale).

## Nutritional screening and assessment of stroke patients

### When and how should nutritional risk and of stroke patients be assessed?

#### Recommendation 10

All stroke patients should be screened for nutritional risk within the first days after hospital admission (CCP).

#### Recommendation 11

Stroke patients at nutritional risk and/or with dysphagia should be assessed more deeply (CCP).

Malnutrition is present in about 24% of stroke patients, with studies reporting prevalences between 8 and 48% depending on patient cohort and assessment technique [56]. Causes for reduced food intake and subsequently impaired nutritional status are various and range from dysphagia to functional disability, impaired consciousness, perception deficits and cognitive dysfunction up to depression [56]. As malnutrition is known to worsen the outcome of various patients groups [57], it should be screened for in stroke patients. The NRS 2002 is the most suitable screening tool for the acute situation of stroke patients, but other screening and assessment tools (i.e. MUST, MNA-LF, MNA-SF, SGA) may as well be applicable [58,59].

## Feeding strategies after stroke

### In what kind of stroke patients can tube feeding improve prognosis?

Ten to thirty percent of all patients after acute stroke are tube fed during the initial phase. It is not yet clear which stroke patients will benefit from tube feeding. Stroke patients with a decreased level of consciousness, severe dysphagia or severe palsy are substantially handicapped in their food intake and are therefore at high risk for malnutrition and likely to benefit from tube feeding. The same is true for patients with severe pre-existing malnutrition.

The second part of the FOOD-trial, including 859 stroke patients, demonstrated a tendency towards a reduced mortality in dysphagic stroke patients by 5.8% (p = 0.09) in the group with early tube feeding, initiated within seven days after stroke [60,61]. It is a limitation of the study that patients were only included when the attending physician was unsure about the adequate nutrition therapy. Hence, patients with a clear indication for early tube feeding were not recruited. The results of this study indicate a potential benefit of an early initiation of tube feeding in such patients, but it remains unclear whether tube feeding may improve prognosis in stroke patients.

### Do patients with a decreased level of consciousness and mechanically ventilated stroke patients profit from tube feeding?

#### Recommendation 12

Patients with a decreased level of consciousness and mechanical ventilation often require enteral nutrition for a longer period of time and tube feeding can therefore start early (C).

There are no systematic trials investigating this issue. Since it is mandatory to artificially feed patients with a relevant decrease of consciousness, it only remains to be decided whether parenteral or enteral nutrition is superior. There are no systematic evaluations comparing parenteral and enteral nutrition in stroke patients. Based on data in other critical care patients, an advantage of tube feeding in stroke patients can be assumed [62] but an influence on mortality has not yet been proven [63].

### Do patients with a presumably long lasting dysphagia profit from enteral nutrition via feeding tube?

#### Recommendation 13

Patients with prolonged severe dysphagia anticipated to last for more than 7 days should receive tube feeding (CCP).

Patients with swallowing difficulties have a high risk for aspiration and aspiration pneumonia as well as for developing malnutrition. Aspiration pneumonia cannot be prevented by tube feeding in the acute phase after stroke [39,64,65]. However, the rate of aspiration pneumonia does not increase during enteral nutrition [60]. Thus, the aspiration risk per se does not represent an indication for tube feeding. But patients with persistent dysphagia are also at risk of malnutrition. Since malnutrition worsens the prognosis and leads to an increased rate of complications, it should be avoided [66,67]. Therefore, patients at risk of prolonged dysphagia should be fed via tube.

### When should nutrition therapy start in stroke patients with swallowing difficulties?

#### Recommendation 14

Severe swallowing difficulties that do not allow sufficient oral food intake and are anticipated to persist for more than 1 week require early enteral nutrition via feeding tube (at least within 72 hours) (C).

From a practical point of view, it is not feasible to start tube feeding on the first day of treatment for most patients, especially in an uncertain situation with possible complications like cerebral hemorrhage or need of ventilation. On the other hand, an early start of enteral nutrition in acute disease does have several advantages: the barrier function of the gut mucosa is kept intact and bacterial translocation of gut-bacteria into the systemic blood flow is thus reduced, leading to less infectious complications with tube feeding compared to parenteral nutrition [68-70].

The only randomized, controlled study evaluating timing of feeding in stroke patients was the “Early versus Avoid Trial” of the FOOD-study [60,61]. After randomisation tube feeding was either started as soon as possible or the placement of the tube was delayed for at least seven days. During this period fluid was given intravenously or subcutaneously. Whether enteral nutrition was given via a percutaneous endoscopic gastrostomy (PEG) or a nasogastric tube, was decided by the attending physician. The group of patients that started enteral nutrition within 7 days of admission had a reduction in mortality by 5.8%, which was not significant (p = 0.09). As the proportion of patients surviving with poor outcome was greater in the group with early nutrition (defined as Rankin Score 4 or 5), it could be speculated that these patients with an “impaired outcome” would have died with a delayed start of nutrition. Pneumonia did not occur more often in patients that received early enteral nutrition. Because this single randomized study (FOOD-trial) has several methodological limitations, the recommendation is graded C.

### Which route of enteral feeding should be preferred?

### What are the indications of a PEG or a nasogastric tube?

#### Recommendation 15

If a sufficient oral food intake is not possible during the acute phase of stroke, enteral nutrition shall be preferably given via a nasogastric tube (A).

#### Recommendation 16

If enteral feeding is likely for a longer period of time (> 28 days), a PEG should be chosen and shall be placed in a stable clinical phase (after 14 – 28 days) (A).

#### Recommendation 17

Mechanically ventilated stroke patients should receive a PEG at an early stage (B).

#### Recommendation 18

If a nasogastric tube is repeatedly removed accidentally by the patient and if artificial nutrition will probably be necessary for more than 14 days, early placement of a PEG should be considered (B). A nasal loop (bridle) is an effective alternative in this situation (B).

Dysphagia due to ischemic cerebral insult resolves within 7–14 days in 73 – 86% of the cases [13,52,71]. It is therefore worthwhile to consider an access to enteral nutrition which is less invasive than percutaneous endoscopic gastrostomy at first. At present, only two prospective, randomized, controlled intervention studies exist that compare nasogastric tube feeding and PEG feeding after stroke.

In a study by Norton et al. that included 30 stroke patients, 16 patients who were assigned to the PEG-group, had a better nutritional status, lower mortality and shorter hospital stay after 6 weeks of intervention [72].

In the FOOD-study no differences between PEG feeding and nasogastric tube feeding could be found regarding the endpoint “death after six months” in 321 dysphagic stroke patients [60,61]. But patients with nasogastric tube feeding showed a significantly 7.8% lower risk of the combined end point “death and/or impaired functional status” when compared to patients with early PEG feeding after 6 months. In addition, there was an increase in pressure sores in the PEG-group (p = 0.04).

In general, dislodgement of nasogastric tubes and by this poor enteral nutrition is a major concern. Two studies about nasal loops in stroke patients demonstrated that nasal loops are safe, well tolerated and effective at delivering full enteral nutrition [73,74]. A recent randomized controlled trial observed an increase of 17% mean volume of fluid and tube feed given in the nasal loop group without any differences in outcome after 3 months [74].

A randomized study published in 2005 by Kostadima et al. reported that early nutrition (within 24 hours) via PEG in 41 mechanically ventilated patients with stroke or head injury was superior to feeding via nasogastric tube, as it was associated with a lower prevalence of ventilator-associated pneumonia [75]. However, a significant difference in length of stay and mortality could not be found. Conclusions for the treatment of ventilated stroke patients can be drawn from this study, as stroke patients were represented with 61%. In particular in mechanically ventilated stroke patients, in whom prolonged artificial nutrition (> 14 days) is probable, early feeding via PEG should be preferred to nasogastric tube feeding, due to a lower rate of ventilation related pneumonia [63,75].

Particular in stroke patients with unfavorable prognosis ethical considerations and supposed will should be considered intensively. In doubt, a semi-invasive nutrition with nasogastric tube feeding might be most appropriate as a potentially reversible first step. The indication for artificial nutrition should be reconsidered daily and in particular thoroughly reassessed before transfer to a nursing home or a palliative-care unit. Tube feeding may be terminated, if the medical indication no longer exists, most likely in a palliative situation. In patients with an uncertain prognosis, PEG-insertion should not be a criterion for the admittance to a rehabilitation ward or to a nursing home, especially not, if a nasogastric tube is well tolerated. The readers may be referred to the latest ESPEN-guidelines “Ethical and Legal Aspects of Enteral Nutrition” [76].

Due to the risk of internal pressure sores, small diameter nasogastric feeding tubes (8 French) should be used in stroke patients. Tubes with a greater diameter should only be placed, when a gastric decompression is necessary. The placement of a nasogastric tube should be done by trained and technically experienced medical staff. Due to the risk of misplacement, the correct position should be controlled before the application of tube feed. This can be done via x-ray or by the aspiration of gastric content and measurement of gastric pH [77]. A local standard for the control of correct tube placement should be developed in every hospital.

### Does duodenal or jejunal tube placement reduces aspiration risk in stroke patients?

#### Recommendation 19

Feeding tubes should be inserted preferably in a gastric position (B).

Gastric tube placement does not present a higher risk for aspiration pneumonia than duodenal or jejunal tube placement. Although this topic has not been investigated in stroke patients, present studies in other patient collectives have not found a significant advantage of post-pyloric tube placement when compared to pre-pyloric placement [78-81].

### Should tube feed be delivered continuously or as a bolus?

#### Recommendation 20

With a previous history of gastroesophageal reflux or when signs of gastroesophageal reflux with aspiration or a high risk of aspiration are present a continuous application of tube feed should be commenced. (B).

#### Recommendation 21

With jejunal or duodenal tube placement, a continuous application is indicated (CCP).

In stroke patients it has not been investigated yet whether or not a continuous feeding leads to lower complication rates or better treatment results. Only one retrospective study from 2002 in 152 patients with traumatic brain damage might be in some aspect transferable to the situation of stroke patients [82]. A significant advantage of continuous feeding concerning feed-tolerance (measured as residual volume >75 ml and bloating) (37.9 vs. 60.5%) and the total number of infectious complications (71.2 vs. 82.6%) was found. The prevalence of pneumonia was not significantly different. There were no differences in functional outcome or nutritional status.

When no risk factors (see above) are present, intermittent bolus application (6 times daily) for respectively 1 hour is just as safe. In particular, patients at high risk for tube dislocation, e.g. agitated patients, who are fed via a nasogastric tube, should be fed with a bolus application distributed in six portions and applied with a syringe. Thereby tube dislocation and following complications can be recognized early.

### Should tube feeds be delivered with a feeding pump or by gravity?

#### Recommendation 22

In stroke patients tube feed should preferably be applied with a feeding pump (CCP).

No data exist regarding this topic in general patients and stroke patients. As dysphagic stroke patients are at significant risk for aspiration, a potentially uncontrolled delivery of tube feed by gravity should be avoided, as this may cause gastric overload and regurgitation with subsequent aspiration.

### Does nasogastric tube feeding interfere with swallowing training and rehabilitation?

#### Recommendation 23

Nasogastric tube feeding does not interfere with swallowing training. Therefore, dysphagia therapy shall start as early as possible also in tube-fed patients (A).

#### Recommendation 24

If there are symptoms of unexplained worsening of dysphagia, the pharyngeal tube position should be controlled endoscopically (B).

Three recent studies, with two of them in stroke patients, did not demonstrate a negative impact of nasogastric tube feeding on swallowing function [83-85]. Dysphagia therapy should therefore start as early as possible, in tube-fed as well as non tube-fed patients. Dziewas et al. demonstrated that in most cases of worsening of dysphagia with a nasogastric tube, this was due to misplacement with coiling of the tube in the pharynx [84]. A reinsertion of the tube or even more favorable an endoscopic evaluation of the pharyngeal tube position is therefore recommended in this situation.

### Should tube-fed patients with dysphagic stroke be advised to have additional oral nutrition?

#### Recommendation 25

The majority of conscious dysphagic stroke patients with tube feeding should have additional oral intake, according to the kind and severity of dysphagia (B).

There is some evidence that tube-fed patients with dysphagic stroke show higher rates of respiratory infections than orally fed patients [3,86]. However, it may be assumed that this is mainly due to higher severity of dysphagia leading to tube feeding. Even patients with nil by mouth have to swallow more than 500 ml of saliva per day and are by this at high risk for aspiration. As aspiration pneumonia is caused by the bacterial content of the saliva and not by the saliva itself [87,88], a strict oral hygiene has the potential of reducing respiratory infections [89,90]. This concept is supported by a study of Gosney et al. that demonstrated a significant preventive effect of selective oral decontamination on the incidence of pneumonia in elderly dysphagic stroke patients [91]. An equal effect of oral decontamination has been shown in the prevention of ventilation associated pneumonia [92].

Although there are no studies in this field, numerous experts recommend minimal amounts of oral intake, such as ice chips in severely dysphagic patients, to promote oral hygiene and the swallowing ability itself. Often dysphagia is present only for certain textures. After thorough clinical or endoscopic evaluation of the safety of different textures, the patient should be fed orally with food of the “safe texture”, to promote oral hygiene and swallowing rehabilitation.

### In which situation is parenteral nutrition indicated in stroke patients?

#### Recommendation 26

Parenteral nutrition is indicated, if enteral nutrition is contraindicated or not feasible (CCP).

#### Recommendation 27

Even in well-nourished patients supplemental parenteral nutrition should be performed if enteral nutrition cannot meet the nutritional needs for more than seven days (CCP).

#### Recommendation 28

If a sufficient hydration by oral or enteral nutrition is not possible, parenteral hydration should be applied immediately (CCP).

There are no data available about parenteral application of energy and nutrition in stroke patients. Indications should be made according to the guidelines on critically ill patients [62].

Some studies have shown inadequate fluid intake of dysphagic stroke patients being placed on an oral diet [93,94]. Accordingly, the fluid intake of dysphagic stroke patients has to be monitored and supplemented, if necessary. This may be done with thickened drinks, intravenous and subcutaneous hydration [93-95]. Especially in the acute phase, peripheral intravenous hydration is most adequate.

### Which patients should receive oral nutritional supplements (ONS, “Sip feeds”)?

#### Recommendation 29

Stroke patients, who are able to eat and who have been identified to be at risk of malnutrition, who are malnourished or who are at risk for pressure sores should receive oral nutritional supplements (B).

In the overall group of stroke patients without dysphagia, oral nutritional supplements do not improve survival or functional outcome. In elderly patients with malnutrition oral nutritional supplements do improve survival. This is probably also true for elderly malnourished stroke patients. In malnourished stroke patients during rehabilitation oral nutritional supplements can support functional recovery. In patients at risk for pressure sores oral nutritional supplements can reduce the rate of pressure sores. This is probably also true for stroke patients.

There are few studies on the effect of ONS in stroke patients. The largest study in this field is the FOOD-trial including 4023 patients [96], which showed no significant influence of ONS on mortality or functional outcome in the entire study collective. However, in 119 undernourished patients who were treated with supplements, a tendency towards better outcome could be seen (mortality or need of assistance: OR 0.78; 95% CI 0.46-1.35; p = 0.39). The data of this trial must be treated with caution as the assessment of nutritional status was not standardized (in 63% of patients only assessed by clinical observation). Furthermore, there were no objective inclusion criteria: patients were only included, when physicians were “uncertain” about the appropriate nutrition therapy and compliance and oral nutritional intake were not recorded. In some smaller studies in acute care and rehabilitation favorable effects of sip feeds on clinical outcome parameters such as functional status and length of stay could be observed [97-99].

A meta-analysis by Milne et al. showed that supplementation reduced the risk of complications (OR 0.86, 95%-CI 0.75 to 0.99) in elderly hospitalized patients. Mortality was unchanged in the overall group, but significantly reduced by oral supplements in elderly hospitalized patients who were malnourished (RR 0.79; 95%-CI 0.64 to 0.97) [100].

In the FOOD-trial the risk for pressure sores was reduced in patients who received sip feeds, but missed significance (p = 0.05) [96]. In other patient groups (patients who did not suffer from acute stroke) sip feeds were associated with a significant reduction (by 25%) in pressure sore development [101]. Extrapolating results from the food trial and meta-analyses [100,101] it is very likely that specific patient groups may profit from oral supplementation. This could be elderly stroke patients who are malnourished when falling ill, who do not show sufficient food intake or who have an increased risk of developing pressure ulcers.

### Is texture modified food or thickened fluid indicated in patients with dysphagia?

#### Recommendation 30

After assessment of the swallowing act (e.g. careful evaluation by the speech-language pathologists and/or videofluoroscopic or endoscopic examination) a texture modified diet and thickened fluids of a safe texture should be given to patients (CCP).

#### Recommendation 31

A dietician should be consulted and nutrition support should be initiated in cases of insufficient intake over a prolonged period of time (C).

In clinical practice textures of fluids and food are modified in order to reduce risk of aspiration, however, there is little research data [102-104]. In a small study in stroke patients Diniz et al. demonstrated that modification of diet texture and thickening of fluids can prevent aspiration in stroke patients [105]. However, patients on texture modified diets tend to have lower nutrient and fluid intakes than patients on a normal diet [93,94].

Clinical or technical evaluation should be the basis for recommendation of the suitable consistency. Depending on the type and severity of the swallowing dysfunction, food should be offered in different consistencies from pureed to soft textures. Dry, stringy or crumbly foodstuff should be avoided as they impair bolus formation. Two-phase food has shown to increase the risk of aspiration [106]. Nectar- or honey thickened consistency of fluids may be helpful in the prevention of aspiration [107].

Studies have shown that patients with dysphagia have an increased risk for malnutrition when compared with patients with intact swallowing function [108]. Patients on texture modified diets have energy and protein intakes which are around 40% lower than that of patients on a normal diet [109]. The same is true for thickened fluids, with studies showing that patients do not meet their fluid requirements [93,94]. Therefore a modification of food texture or a thickening of fluids should only be used after assessment and be monitored by specialized staff (e.g. speech-language therapists and dietician).

## Abbreviations

CBA: Clinical bedside assessment; FEES: Fiberendoscopic evaluation of swallowing; FOOD: Feed or ordinary diet; ONS: Oral nutritional supplements; PEG: Percutaneous endoscopic gastrostomy; VFSS: Video fluoroscopic swallowing study; WST: Water swallowing test.

## Competing interests

Consultancy for a company: R. Dziewas, R. Wirth.

Honoraria for lectures: R. Dziewas, C. Smoliner, T Warnecke, M. Jäger, A. Leischker, R. Wirth.

Research grants: R. Dziewas, T Warnecke, R. Wirth.

## Authors’ contributions

RD, CS and RW have written this manuscript. AL, MJ and TW were active members of the guideline-working group “Clinical Nutrition in Neurology” and reviewed this manuscript. All authors read and approved the final manuscript.
